# Transcriptional activation of glucose transporter 1 in orthodontic tooth movement-associated mechanical response

**DOI:** 10.1038/s41368-018-0029-7

**Published:** 2018-08-15

**Authors:** Yu Wang, Qian Li, Fuliang Liu, Shanshan Jin, Yimei Zhang, Ting Zhang, Yunyan Zhu, Yanheng Zhou

**Affiliations:** 1 Department of Orthodontics, Peking University School and Hospital of Stomatology, Beijing, China; 2 National Engineering Laboratory for Digital and Material Technology of Stomatology, Peking University School and Hospital of Stomatology, Beijing, China; 3 Beijing Key Laboratory of Digital Stomatology, Peking University School and Hospital of Stomatology, Beijing, China

## Abstract

The interplay between mechanoresponses and a broad range of fundamental biological processes, such as cell cycle progression, growth and differentiation, has been extensively investigated. However, metabolic regulation in mechanobiology remains largely unexplored. Here, we identified glucose transporter 1 (GLUT1)—the primary glucose transporter in various cells—as a novel mechanosensitive gene in orthodontic tooth movement (OTM). Using an in vivo rat OTM model, we demonstrated the specific induction of Glut1 proteins on the compressive side of a physically strained periodontal ligament. This transcriptional activation could be recapitulated in in vitro cultured human periodontal ligament cells (PDLCs), showing a time- and dose-dependent mechanoresponse. Importantly, application of GLUT1 specific inhibitor WZB117 greatly suppressed the efficiency of orthodontic tooth movement in a mouse OTM model, and this reduction was associated with a decline in osteoclastic activities. A mechanistic study suggested that GLUT1 inhibition affected the receptor activator for nuclear factor-κ B Ligand (RANKL)/osteoprotegerin (OPG) system by impairing compressive force-mediated RANKL upregulation. Consistently, pretreatment of PDLCs with WZB117 severely impeded the osteoclastic differentiation of co-cultured RAW264.7 cells. Further biochemical analysis indicated mutual regulation between GLUT1 and the MEK/ERK cascade to relay potential communication between glucose uptake and mechanical stress response. Together, these cross-species experiments revealed the transcriptional activation of GLUT1 as a novel and conserved linkage between metabolism and bone remodelling.

## Orthodontics: Glucose transport needed to straighten teeth

A glucose-transporting protein is key to helping teeth respond to orthodontic implants, say researchers in China. Implants apply forces to teeth and the periodontal ligament (PDL) that holds them in place, causing bone to grow on one side and be absorbed into the body on the other. Yanheng Zhou and co-workers at Peking University in Beijing showed that GLUT1, a protein that transports glucose through cell membranes, was greatly upregulated in rat, mouse and human PDL cells subjected to mechanical force. They also injected some of the mice with a GLUT1 inhibitor and found that the treatment greatly decreased the distance moved by the teeth. This could be attributed to a decline in the activity of cells that break down bone tissue and a failure in signalling channels when GLUT1 is inhibited.

## Introduction

The tissue microenvironment, including the extracellular matrix and three-dimensional geometrics, imposes physical constraints on solid tissues.^1,2^ These physical cues affect numerous cellular processes, such as cell differentiation and proliferation, through widespread crosstalk with various signalling cascades.^3–7^ Despite the vital importance of metabolism to life, little is known about its interaction with mechanoresponses. However, limited evidence can still provide a glimpse into the significance of metabolic regulation in mechanobiology. For example, mechanical force can regulate the metabolic pathways in hepatic stellate cells and skeletal muscles.^8,9^

Glucose is the primary source of energy for most cells in our body. The highly conserved glucose transporter 1 (GLUT1), as a member of the major facilitator superfamily of membrane transporters, mediates the transport of glucose across the plasma membrane and shows ubiquitous expression patterns in many cell types.^10^ Interestingly, GLUT1 has been recently identified as the glucose transporter of osteoblast cells with important functions in regulating glucose metabolism and bone homoeostasis.^11^ Another study reported the significance of GLUT1 in insulin-like growth factor  (IGF)-1-mediated promotion of bone formation in diabetic rats.^12^ Meanwhile, the dominant expression of GLUT1 in the erythrocyte membrane is critical for the regulation of osteoclast differentiation and osteoclastic resorption.^13^

In a typical mechanical response, mechanotransduction in orthodontic tooth movement (OTM) would induce bone resorption on the compression side and bone formation on the tension side of the periodontal ligament (PDL).^14^ The PDL is composed primarily of extracellular matrix components and PDL cells (PDLCs), which together coordinate the balance between bone formation and resorption.^15^ PDLCs are able to select and attract osteoclast precursors, and the cytokines produced by PDLCs stimulate the differentiation of osteoclast precursors towards mature osteoclasts, with the receptor activator for nuclear factor-κ B Ligand (RANKL)-receptor activator for nuclear factor-κ B (RANK)-osteoprotegerin (OPG) axis playing crucial roles in this process.^16^

Interestingly, although few studies have investigated the expression and functionality of GLUT1 in PDLCs, abnormal changes in glucose levels have been shown to influence the functions of PDLCs, such as an accumulation of extracellular matrices and integrins, as well as PDLC cell attachment.^17,18^ Furthermore, several studies have uncovered the link between blood glucose levels and OTM rates.^19–21^ Together with the essential role of GLUT1 in the maintenance of bone homoeostasis, it is plausible to speculate that GLUT1, as one of the most important glucose transporters, might play key roles in regulating PDLCs’ various functions, including mediating OTM.

In this study, we use a combination of OTM models both in vivo and in vitro to demonstrate the novel and universal upregulation of GLUT1 by mechanical stimuli. We further evaluated the biological response to this transcriptional activation event and dissected the underlying molecular mechanisms. These results together highlight the significance of the transcriptional activation of GLUT1 as a key component linking metabolism and bone homoeostasis.

## Results

### Mechanical force upregulates GLUT1 expression in PDLCs

**Fig. 1:**
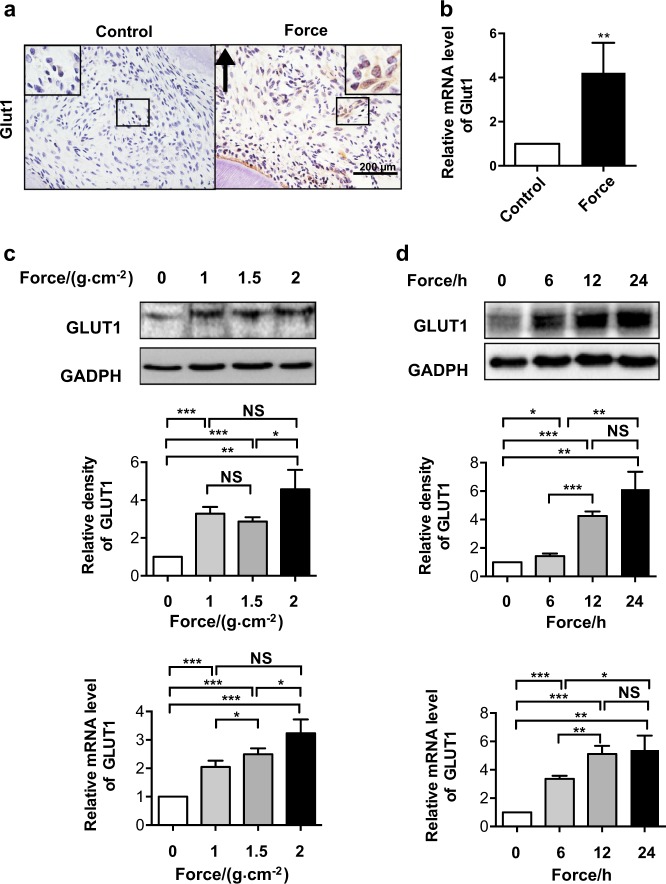
Mechanical force upregulates glucose transporter 1 (GLUT1) expression in periodontal ligament cells (PDLCs). **a, b** Expression of Glut1 increased after orthodontic force application in rats. **a** Representative immunohistochemical images of Glut1 on the compression side of distal roots subjected to orthodontic force or the control side without force application in rats. Large boxed areas show higher magnification views of the small boxes. The arrow indicates the direction of orthodontic force. Scale bars: 200 µm. **b** Relative RNA expression of GLUT1 was determined by quantitative reverse-transcription polymerase chain reaction (qRT-PCR) analysis using mRNA extracted from PDL on the compression side of distal roots subjected to orthodontic force or the control side without force application in rats. *n* = 4–5/group. **c, d** Mechanical force-induced activation of GLUT1 in human PDLCs. **c** Expression changes of GLUT1 at protein and RNA levels in PDLCs treated with increasing force intensity for 24 h were determined by western blot (top and middle) and qRT-PCR (bottom), respectively. **d** Expression changes of GLUT1 at protein and RNA levels in PDLCs treated with varying durations at 1.5 g·cm^−2^ force were determined by western blot (top and middle) and qRT-PCR (bottom), respectively. GAPDH served as a loading control. Data represent mean ± SD. *P < 0.05; **P < 0.01; ***P < 0.001; NS, not significant (*P* > 0.05)

In our attempt to identify novel mechanosensitive genes, we found that the critical glucose transporter GLUT1 was potentially linked to mechanoresponse in a pilot experiment. To validate this point in an in vivo setting, we established a rat OTM model by connecting the maxillary first molar to incisors with a consistent force of ~60 g (Fig. 1a). Histopathological examination of strained rats revealed a concentrated Glut1 staining pattern along the boundary of the PDL and indicated a large fraction of cells with significantly induced Glut1 expression spreading in the area where the compressive force was applied; however, these patterns were not detected in the unstrained control PDL (Fig. 1a). Quantitative reverse-transcription polymerase chain reaction (qRT-PCR) analysis of the mRNA levels of Glut1 in the OTM model suggested that mechanical force-mediated GLUT1 upregulation occurred at the transcription level (Fig. 1b).

To strengthen this observation, we purified PDLCs from human samples using established procedures^22^ and examined expression changes of GLUT1 following physical stress. Consistently, the in vitro cultured PDLCs showed dose-dependent transcriptional activation of GLUT1 at both protein and mRNA levels (Fig. 1c). Time course examination of the mechanoresponses of GLUT1 also showed a gradual increase in GLUT1 at the protein and mRNA levels as the force application time continued (Fig. 1d). These in vivo and in vitro data collectively confirmed the mechanosensitivity of GLUT1.

### GLUT1 inhibition suppressed OTM in mice

**Fig. 2:**
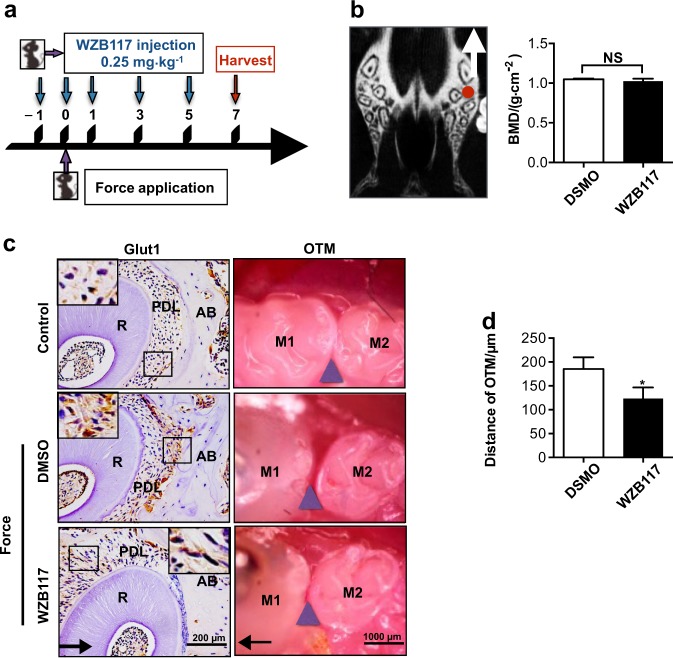
Distance of orthodontic tooth movement (OTM) decreased upon injection of GLUT1 inhibitor WZB117 in mice. **a** Schedule diagram of the experiment. Orthodontic force was applied to mice in two groups for seven days. Injection of DMSO or WZB117 was performed every other day starting on 1 day prior to the 7-day course of OTM. The DMSO-injected control group included seven mice, and the WZB117-injected group included 8 mice. **b** WZB117 injection did not significantly influence the bone mineral density (BMD) of mice. The BMD of alveolar bone was determined in the furcation area (red dot in the left panel) of the upper first molar from the control side without force application in mice treated as in **a**. *n* = 4 /group. The white arrow indicates the direction of orthodontic force applied. **c**–**e** Injection of WZB117 suppressed the upregulation of Glut1 caused by orthodontic force application, and reduced OTM distance. **c** Representative images of the occlusal view of the first and second molars in the two groups of mice treated as in **a** or in untreated control mice with the representative immunohistochemical images of Glut1 in each group shown on the left. Large boxed areas show higher magnification views of the small boxes. Arrows indicate the direction of orthodontic force. Scale bars: 200 µm. **d** Statistical analysis of the OTM distance measured from images as in **c**. *n* = 6. Data represent mean ± SD. *P < 0.05; **P < 0.01; ***P < 0.001. NS, not significant (*P* > 0.05)

To test whether the transcriptional activation of GLUT1 played an active role in periodontal tissue remodelling, we performed a loss-of-function experiment to explore the effect of GLUT1 inhibition on tooth movement (Fig. 2a). Mice were injected with WZB117, a small-molecule inhibitor of GLUT1, or the vehicle, DMSO. WZB117 was reported to be capable of associating with GLUT1, thereby potently inhibiting its activity. Additionally, the protein level of GLUT1 was also found to be downregulated by WZB117.^23^

Scanning the furcation region of the first maxillary molar with micro-computed tomography, we confirmed that there was no discernible difference between WZB117-injected mice and vehicle-injected mice in bone marrow density (BMD), excluding the possibility that WZB117 might alter the process of OTM by affecting alveolar bone density in mice (Fig. 2b).

Immunohistochemical staining confirmed Glut1 elevation upon force application and Glut1 inhibition by injection of WZB117 (Fig. 2c). OTM was then measured as the distance between the midpoint of the distal-marginal ridge of the first molar and the midpoint of the mesial-marginal ridge of the second molar. Interestingly, the WZB117-treated mice showed significantly decreased OTM distance under physical strain compared to the DMSO-treated group (Fig. 2c, d).

### Inhibition of GLUT1 represses osteoclast differentiation in vivo and in vitro

**Fig. 3:**
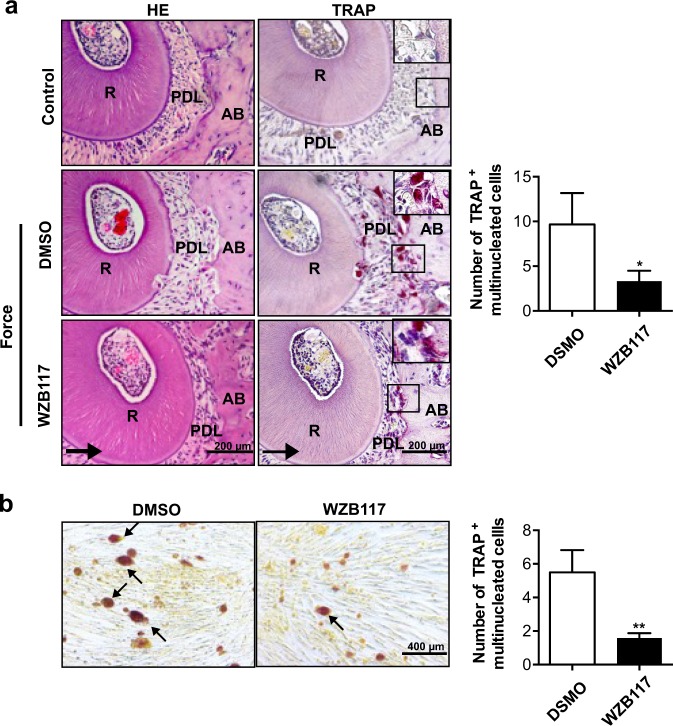
Inhibition of GLUT1 by WZB117 repressed osteoclastic activity during OTM in vivo, and osteoclast differentiation of RAW264.7 cells in vitro. **a** Injection of WZB117 reduced the number of tartrate-resistant acid phosphatase (TRAP)-positive cells during OTM in mice. TRAP staining and haematoxylin and eosin (H&E) staining on the compression side of the distal root of the upper first molar in untreated control mice or in mice treated as in Fig. 2a. Large boxed areas show higher magnification views of the small boxes. Arrows show the direction of orthodontic force. Scale bar: 200 µm. **b** WZB117 treatment in PDLCs prohibited osteoclast differentiation of co-cultured RAW264.7 cells. TRAP staining of osteoclasts in RAW264.7 cells co-cultured with PDLCs pretreated with DMSO or WZB117 followed by osteoclast induction by the addition of sRANKL. Arrows indicate TRAP-positive multinucleated cells in each image. Scale bar: 200 µm. Data represent mean ± SD. *n* = 6, *P < 0.05; **P < 0.01

Since physical strain-mediated OTM functionally requires osteoclastogenesis at the compression side along the direction of applied force,^24^ we then used tartrate-resistant acid phosphatase (TRAP) staining to evaluate the regulation of TRAP-positive osteoclasts by GLUT1 during OTM. Consistently, we observed a significant decline of TRAP-positive osteoclasts on the compression side of PDL and alveolar bone in WZB117-treated mice compared to the control group (Fig. 3a).

To directly validate the importance of force-induced GLUT1 in osteoclast differentiation, we used a co-culture system consisting of in vitro purified PDLCs and osteoclastic precursors of the monocyte/macrophage lineage RAW264.7 cells.^25,26^ As before, the PDLCs were treated with WZB117 or DMSO before their co-culturing with RAW264.7 cells. Microscopic inspection of TRAP staining gave a consistent result, which showed fewer TRAP-positive multicoated cells when the PDLCs were pretreated with WZB117 instead of DMSO (Fig. 3b, *P* < 0.01, two-sided *t*-test). These results collectively suggested an active role of GLUT1 in mechanical stress-induced OTM and osteoclastogenesis.

### Regulation of RANKL/OPG ratio by GLUT1 in PDLCs

**Fig. 4:**
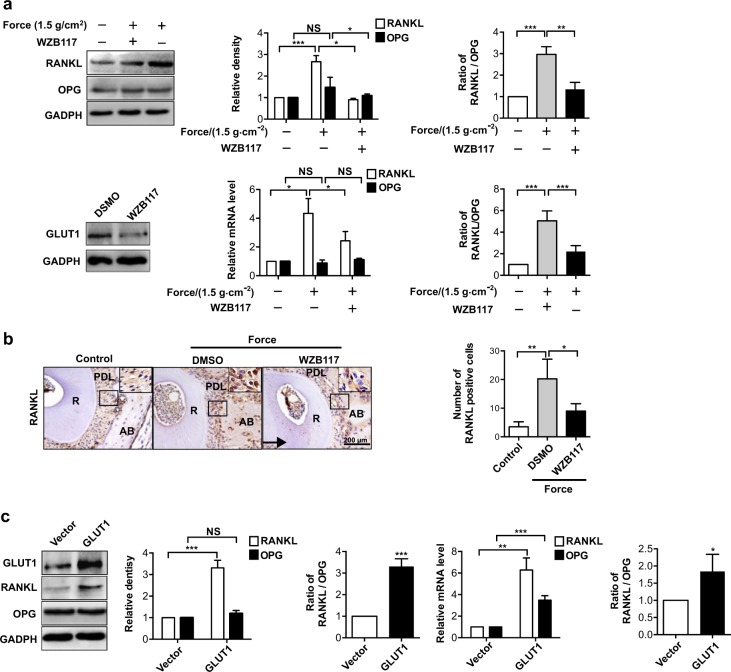
Force-induced GLUT1 expression regulates receptor activator of nuclear factor–κB ligand (RANKL)/osteoprotegerin (OPG) ratio in PDLCs. **a** WZB117 inhibition of GLUT1 decreased force-induced upregulation of RANKL/OPG ratio. Protein and mRNA levels of RANKL and OPG were determined in control PDLCs or cells subjected to mechanical force or together with WZB117 treatment. The bottom panel shows inhibited expression of GLUT1 when WZB117 was administered at concentrations of 10 μmol·L^−1^. Ratios of RANKL/OPG were calculated based on relative protein or RNA levels of RANKL and OPG. **b** Injection of WZB117 decreased RANKL activation during OTM in mice. Immunohistochemical analysis of RANKL expression on the compression side of distal roots in untreated control mice or in mice treated as in Fig. 2a. Large boxed areas show higher magnification views of the small boxes. The arrow shows the direction of orthodontic force. Scale bar: 200 µm. **c** Overexpression of GLUT1 upregulated RANKL/OPG ratio in PDLCs. Protein and RNA expression levels of RNAKL and OPG were determined in PDLCs transfected with plasmid overexpressing GLUT1 or its control vector. Data represent mean ± SD. *P < 0.05; **P < 0.01; *** *P* < 0.001; NS, not significant (*P* > 0.05)

We next sought to determine the molecular mechanisms underlying GLUT1-mediated mechanoresponses and regulation on bone homoeostasis during OTM. OPG shows competitive inhibitory binding to RANK to block RANK’s association with its natural ligand RANKL in bone resorption,^27,28^ and compressive force could alter the expression balance between OPG and RANKL to regulate osteoclastogenesis.^16^ Thus, we first determined the expression patterns of RANKL and OPG during OTM in the presence or absence of GLUT1 inhibitor WZB117. Western blotting analysis indicated that the DMSO-treated PDLCs showed robust induction of RANKL; however, when the PDLCs were pretreated with WZB117, the mechanoresponses of RANKL were significantly suppressed (Fig. 4a). qRT-PCR analysis gave a similar trend of WZB117’s effect on the mRNA levels of RANKL (Fig. 4a). On the other hand, the treatment of WZB117 had no significant effect on OPG expression (Fig. 4a). This point was further confirmed in an in vivo mouse OTM model, where force-induced RANKL expression on the compression side of the PDL was evidently suppressed by WZB117 treatment compared to the control group (Fig. 4b).

Interestingly, the overexpression of GLUT1 could elicit a comparable induction of RANKL expression at both protein and mRNA levels without any mechanical stress applied, implying the sufficiency of GLUT1-mediated glucose uptake for the transmission of mechanosignals during OTM (Fig. 4c).

### Functional interaction between GLUT1 and ERK pathway in mechanotransduction

**Fig. 5:**
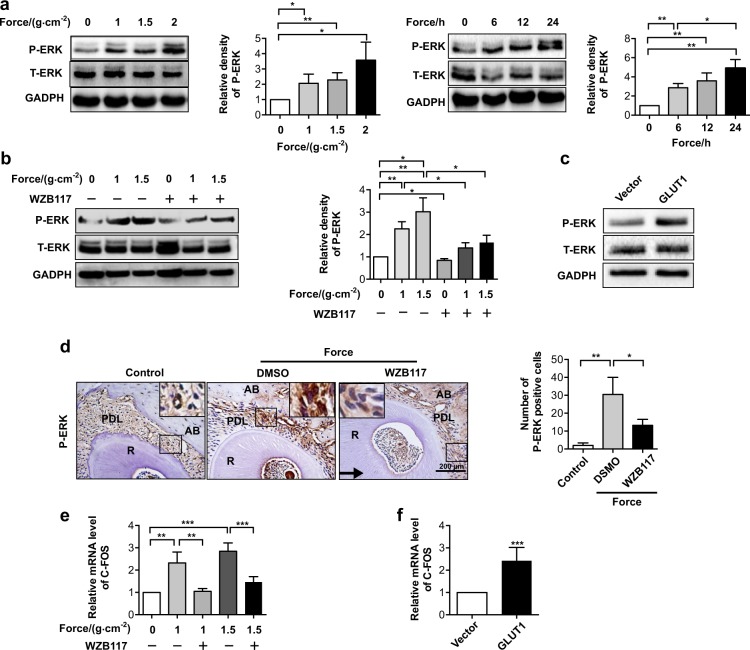
Force-induced activation of ERK partially depends on GLUT1 activation. **a** ERK phosphorylation increased in PDLCs exposed to mechanical force. Expression changes of phosphorylated ERK (P-ERK) were determined in PDLCs treated with increasing force intensity for 24 h (left) or varying durations at 1.5 g·cm^−2^ force (right). **b** Inhibition of GLUT1 attenuated force-induced elevation of phosphorylated ERK. Protein expression of phosphorylated or total ERK (P-ERK and T-ERK, respectively) was determined in PDLCs treated with indicated force intensity or in combination with WZB117 treatment. **c** Overexpression of GLUT1 upregulates P-ERK. Protein expression of P-ERK and T-ERK was determined in PDLCs transfected with plasmid overexpressing GLUT1 or its control vector. GAPDH served as a loading control. **d** Injection of WZB117 decreased P-ERK activation during OTM in mice. Immunohistochemical analysis of P-ERK expression on the compression side in mice treated with orthodontic force and injected with WZB117 or DMSO as a control. Large boxed areas show higher magnification views of the small boxes. The arrow indicates the direction of orthodontic force. **e, f** WZB117 administration suppressed force-induced c-FOS activation, whereas overexpression of GLUT1 promoted c-FOS expression. **e** mRNA expression of c-FOS was determined in PDLCs treated with indicated force intensity or in combination with WZB117 treatment. **f** mRNA expression of c-FOS was determined in PDLCs transfected with GLUT1 plasmid or its control vector. Data represent mean ± SD. *P < 0.05; **P < 0.01; *** *P* < 0.001; NS, not significant (*P* > 0.05)

To unveil the functional interaction between GLUT1-mediated nutrient assimilation and physical strain-elicited stress response in PDLCs, we focused on MAPK/ERK cascades. This cascade was selected because ERK phosphorylation could be activated by mechanical stimulus in PDLCs,^29,30^ and ERK was involved in the transcription regulation of RANKL/OPG and required for osteoclastogenesis.^31–33^ Meanwhile, a glucose-mediated activation effect on ERK was found to be amplified in GLUT1-overexpressed cells.^34^ Therefore, we then tested whether mechanical force-induced GLUT1 upregulation could contribute to ERK activation in PDLCs. To this end, PDLCs were pretreated with WZB117 or vehicle before their exposure to compressive force. Western blotting results showed that this physical stress successfully led to ERK activation as indicated by elevated phosphorylation in the control cells, and importantly, this effect was partially rescued by WZB117 treatment, suggesting that GLUT1 inhibition impaired ERK-mediated mechanosignalling (Fig. 5a, b). Moreover, administration of WZB117 in the mouse model greatly suppressed physical strain-induced ERK phosphorylation in vivo (Fig. 5d). Consistently, overexpression of GLUT1 induced robust upregulation of ERK phosphorylation in the absence of mechanical stress (Fig. 5c). As a classical downstream target gene of ERK, c-FOS was reported to be a crucial player during osteoclast differentiation;^35,36^ we then evaluated whether GLUT1 could influence c-FOS expression. Quantitative expression analysis suggested that when mechanical stress-mediated GLUT1 upregulation was inhibited by WZB117, transcriptional activation of c-FOS was significantly suppressed (Fig. 5e). Furthermore, ectopic expression of GLUT1 was sufficient to activate c-FOS expression (Fig. 5f).

**Fig. 6:**
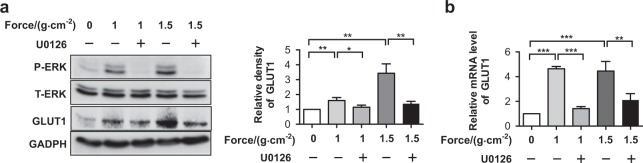
Activation of ERK1/2 phosphorylation was required for force-induced GLUT1 upregulation in PDLCs. Administration of U0126 weakened the force-induced activation of GLUT1. Protein **a** and mRNA **b** levels of GLUT1 were determined in PDLCs subjected to mechanical force with indicated intensity or in combination with the administration of U0126. Data represent mean ± SD. *P < 0.05; **P < 0.01; ***P < 0.001

On the other hand, according to the reports that ERK activation occurs as an upstream event for GLUT1 activation in cells stressed with various stimuli,^37,38^ it would be interesting to test whether ERK activation was essential for GLUT1 upregulation in mechanoresponses. We thus suppressed ERK activation in PDLCs by using the MEK inhibitor U0126.^39^ We found that U0126 treatment caused a decline in physical stress-mediated GLUT1 induction at both protein and mRNA levels in PDLCs (Fig. 6). These results suggested that a mutual regulation schema between GLUT1 and the MAPK/ERK cascade exists for mechanical stress-mediated metabolic regulation and stress response.

## Discussion

In this study, we demonstrated that mechanical force could induce GLUT1 expression in both an in vivo OTM model and in vitro cultured human PDLCs stressed with compressive force, and this regulation contributed to osteoclast differentiation in periodontal tissue remodelling. This conclusion was drawn from the following facts: first, mechanical force elicited evolutionarily conserved upregulation of GLUT1 in PDL; second, pharmacological inhibition of GLUT1 by WZB117 led to reduced OTM and osteoclastic activities; third, force-induced GLUT1 expression in PDLCs affected the RANKL/OPG ratio to regulate osteoclast differentiation; and finally, GLUT1 functionally interacted with the MAPK/ERK cascades in mechanoresponses. Our conclusion is well supported by the identification of GLUT1 as the major glucose transporter in osteoblasts.^40^ Furthermore, the expression of GLUT1 was enhanced in various cells and tissues in response to hypoxia.^41,42^ Since mechanical force can create a hypoxic environment on the compression side of the periodontium during OTM,^43^ the observation of hypoxia-induced GLUT1 activation supported our point. However, to the best of our knowledge, this study is the first work to show that GLUT1 is a mechanosensitive gene in periodontal tissue, and this result gives novel insight into mechanosignalling.

In line with our observations that force-induced GLUT1 influences osteoclast differentiation in PDL, the implications of GLUT1 in bone resorption have been observed in other conditions. Diabetes mellitus studies have shown that type 2 diabetes rats with greater GLUT1 expression exhibited greater OTM and increased RANKL expression;^20,21^ and hyperglycaemia has been found to suppress RANKL-induced osteoclast differentiation.^19,44^ In addition, cancer cells under high glucose concentrations showed reduced GLUT1 and HIF1α expression.^45^ The convergence of GLUT1 and osteoclastogenesis could also be attributed to the hypoxic stress created by physical strain in PDL, where enhanced RANKL expression could be observed along with the hypoxic environment in human PDLCs.^46,47^ Our mechanistic study established a causal relationship between GLUT1 and RANKL expression through both gain-of-function and loss-of-function experiments, thus providing the molecular basis for GLUT1-enhanced osteoclastogenesis during OTM. Indeed, periodontal tissue remodelling during OTM is initiated with bone resorption owing to osteoclastogenesis—a rate-limiting step for tooth movement—and requires its coordination with osteoblast-mediated bone formation, thereby demanding abundant nutrients for this tissue’s regeneration. Similarly, a recent study found that glucose uptake, by means of the facilitative GLUT1 glucose transporter in osteoblasts, is necessary for differentiation and subsequent bone formation.^11^ Therefore, our data linked GLUT1’s contribution to osteoclastogenesis in OTM.

Previous observations that mechanosensitive ERK phosphorylation was implicated in cytokine-mediated signalling pathways during differentiation, activation or migration of osteoclasts and that overexpression of GLUT1 could amplify glucose-mediated ERK activation led us to test the possibility that GLUT1 might play a role during force-induced ERK activation. As expected, GLUT1 inhibition by WZB117 attenuated phosphorylated ERK elevation upon force stimuli, which might provide another mechanism for GLUT1-mediated clastogenesis during OTM. In addition to its influence on RANKL/OPG, force-induced GLUT1 may contribute to the activation of ERK, which further promotes osteoclast differentiation and ultimately OTM rate. Interestingly, ERK activation and GLUT1 upregulation in PDL are interdependent, as compressive force-induced GLUT1 expression in PDLCs could be reverted by using the MEK inhibitor U0126. Similarly, ERK activation was evidenced to be involved in the upstream events of GLUT1 in the angiotensin II signalling scenario in diabetic nephropathy.^34,37^ The interplay between ERK and GLUT1 is reasonable and could occur at many levels due to the extensive functional or physical interactions between RAF/MAPK/ERK kinases with a wide range of scaffold proteins from other signalling cascades.^48,49^ Hence, mutual regulation between GLUT1 and ERK could affect various stages during OTM.

In summary, our results highlighted an intriguing mechanism by which OTM is regulated by the force-induced transcriptional activation of GLUT1, thus providing an improved understanding of mechanoresponses from a metabolic perspective.

## Materials and Methods

### Animals and orthodontic force application

For the rat OTM experiment, 15 Sprague-Dawley rats (male, 8-week-old, Weitong Lihua Experimental Animal Center, Beijing, China) weighing 180 g to 220 g were used. For tooth movement, we used a nickel-titanium coil spring (wire size, 0.2 mm; diameter, 1 mm; length, 4 mm; Smart Technology, Beijing, China) to connect the maxillary left first molar to incisors, which can provide a constant force of ~60 g. The contralateral first molar served as the control.

For the mouse OTM experiment, C57BL/6 mice (male, 6-8-week-old, Weitong Lihua Experimental Animal Center, Beijing, China) weighing 20 g to 25 g each were used. The method of application of mechanical force was modified on basis of the previously described approach.^50^ Briefly, a nickel-titanium coil spring (wire size, 0.2 mm; diameter, 1 mm; length, 4 mm; Smart Technology, Beijing, China) was bonded between the maxillary right first molar and maxillary incisors by flowable restorative resin (3 M ESPE, USA) to deliver a force of 30 g. The occlusal surface of the contralateral first molar was covered with resin to serve as the control.

All of the rat and mouse experimental protocols were approved by the Institutional Animal Care and Use Committee of Peking University (LA2013-92).

### Experimental treatment grouping

Mice were randomly divided into two groups (*n* = 15), including an experiment group and a vehicle group. Both groups received orthodontic force application. The experiment group was injected with a specific GLUT1 inhibitor, WZB117 (S7927, Selleck).^23^ For systemic administration of WZB117, each mouse was injected intraperitoneally with WZB117 solution at 10 mg·kg^−1^. The WZB117 solution was prepared by dissolving in dimethylsulfoxide (DMSO) (40 mg·mL^−1^ in stock) and diluting in PBS to form 200 μL solutions for each injection. At the same time, all mice in the vehicle group received an injection of an equal volume of DMSO diluted in PBS. Mice were first injected one day before the application of mechanical force. Then, injections were performed every other day until the last day of force application.

### Measurement of OTM distance and micro-computed tomography analysis

After sacrificing the mice using a pentobarbital sodium overdose, we obtained the maxillae. Then, we used a stereo microscope (SWZ1000, Nikon, Japan) to record the occlusal surface of the maxillae. The OTM distance is measured between two easily located points (the midpoint of the distal-marginal ridge of the first molar and the midpoint of the mesial-marginal ridge of the second molar). Two researchers who were blinded to the entire experimental process measured the OTM distance using ImageJ 1.37v software (Wayne Rasband).

Micro-computed tomography (Skyscan1174, Bruker micro CT, Belgium) was used to scan the maxillae of mice. To analyse the BMD, we choose the furcation of the first molar as the measurement area due to its reproducible morphological landmarks.^51^

### Immunohistochemical staining

We performed immunohistochemical staining experiments with a two-step detection kit (Zhongshan Golden Bridge Biotechnology, Beijing, China) as previously described.^52^ The primary antibodies used were anti-GLUT1 (1:100, 21829-1-AP, Proteintech), anti-RANKL (1:100, ab45039, Abcam), and anti-phospho-ERK (1:100, 4370, Cell Signaling Technology).

### Histology and Eosin (H&E) staining and TRAP staining

Maxillae samples of rats and mice were collected, fixed in 4% paraformaldehyde, demineralised in 15% EDTA (ethylenediaminetetraacetic acid), and then embedded in paraffin. A transverse cutting method was employed to obtain serial sections from the corresponding group at a thickness of 4 μm. Then, the sections were deparaffinized and used to perform H&E staining to describe the histological characteristics. TRAP staining was performed with an acid phosphatase kit (387A-1KT; Sigma, USA) following the manufacturer’s instructions. We chose the area attached to the alveolar bone in the PDL to count the number of TRAP-positive cells.

### Cell lines, cell culture and treatments

The protocols to obtain the extracted teeth were approved by the Ethical Guidelines of Peking University. We conducted the protocol with appropriate informed consents (PKUSSIRB-201311103). Human PDLCs were isolated from the normal orthodontic extracted bicuspids as previously reported. PDLCs were cultured in α-MEM supplemented with 10% foetal bovine serum (FBS), 0.292 mg·mL^−1^ glutamine, 100 units per mL penicillin streptomycin, and 100 mmol·L^-1^ ascorbic acid and incubated at 37 °C in a humidified atmosphere containing 5% CO_2_. Cells were used at 3–4 passages. For the co-culture experiment, the murine macrophage RAW264.7 cell line was cultured in α-MEM containing 10% FBS.

Static compressive force was applied on PDLCs as previously described. Briefly, a layer of glass cover and corresponding metal balls on top were placed over the cell layer, which was 80% confluent in 6-well plates. Then, PDLCs were exposed to different static compressive forces ranging from 0 to 2 g·cm^−2^ for 24 h or at 1.5 g·cm^−2^ for varying durations ranging from 0 to 24 h.

To inhibit the expression of GLUT1 in PDLCs, WZB117 was added into the medium with a final concentration of 10 μmol·L^-1^. For the control cells, DMSO with the same volume was added.

To achieve GLUT1 overexpression, the plasmid pcDNA3.2/v5-DEST GLUT1 and the control vector pcDNA3.2 (purchased from Addgene) were transfected, respectively, into PDLCs using the Lipotectamine Stem Transfection Reagent (STEM00001) according to the protocol provided by the manufacturer. The cells were selected through the addition of G418 into the medium, and stable cell lines were generated.

For the inhibition of ERK phosphorylation, PDLCs were treated with MEK-specific inhibitor U0126 (final concentration: 1 μmol· L^−1^) for 24 h prior to mechanical force stimulation.^53^

### Co-culture of PDLC and RAW264.7 and TRAP staining

PDLCs were seeded into 12-well plates at 5 × 10^3^ cells per well and treated with WZB117 or DMSO for one day. Then, the medium was replaced and RAW264.7 cells were added to the well at 5 × 10^5^ cells/well. sRANKL (50 ng· mL^−1^) was added for the induction of osteoclast differentiation. The medium was semi-changed every three days. After seven days, the cells were fixed and stained for TRAP staining using an acid phosphatase kit (387A-1KT; Sigma, USA). TRAP-positive multinucleated (>2 nuclei) osteoclasts were counted in 5 visual fields at ×20 magnification in each well (*n* = 3). We calculated the average value of three experiments.

### Western blotting analyses and antibodies

Western blotting analyses were performed according to protocols described previously.^54^^,^^55^ The antibodies used in this study are as follows: anti-GLUT1 (21829-1-AP, Proteintech); anti-RANKL (ab45039, Abcam); anti-osteoprotegerin (OPG) (ab11994, Abcam); anti-phospho-p44/42 MAPK (ERK1/2) (Thr202/Tyr204) (4370, Cell Signaling Technology); anti-p44/42 MAPK (ERK1/2) (4695, Cell Signaling Technology); and anti- glyceraldehyde-3-phosphate dehydrogenase (GAPDH) (sc-47724, Santa Cruz). Each experiment was repeated three times. The relative density of results was measured by ImageJ 1.37 v software.

### Quantitative reverse-transcription polymerase chain reaction (qRT-PCR)

Total RNAs were isolated from cultured PDLCs or periodontal tissue of rats using Trizol reagent according to the manufacturer’s instructions. Reverse-transcription and real-time PCR were performed following protocols described elsewhere.^55,56^ All qRT-PCR processes were performed three times using GAPDH as the internal control. The primers (designed by Primer3 Plus online) used in this study are as follows: human (h) GAPDH forward (F): caatgaccccttcattgacc, hGAPDH reverse (R): atgacaagcttcccgttctc; hRANKL F: ATCACAGCACATCAGAGCAGAGA, hRANKL R: AGGACAGACTCACTTTATGGGAAC; hOPG F: gaggcattcttcaggtttgc, hOPG R: gctgtgttgccgttttatcc; Rat(r) Glut1 F: ttctccaaactgggcaagtc, rGlut1 R: cacatacatgggcacaaagc; r gapdh F: ctcatgaccacagtccatgc, r gapdh R: ttcagctctgggatgacctt; and h c-FOS F: aacctgtcaagagcatcagc, h c-FOS R: atgatgctgggaacaggaag.

### Statistical analysis

SPSS 19.0 was used to perform statistical analysis. All data were presented as the mean ± SD and assessed by independent two-tailed Student’s *t*-test or One-way analysis of variance (ANOVA). Tukey’s multiple-comparison test was used for the post hoc comparison of ANOVA. Differences with *P* < 0.05 were considered statistically significant.
